# Functional Connectivity Linked to Cognitive Recovery After Minor Stroke

**DOI:** 10.1002/acn3.70271

**Published:** 2025-12-06

**Authors:** Vrishab Commuri, Isabella Dallasta, Ciaran Stone, Sophia Girgenti, Neda Gould, Rafael H. Llinas, Jonathan Z. Simon, Elisabeth Breese Marsh

**Affiliations:** ^1^ Department of Electrical and Computer Engineering The University of Maryland College Park Maryland USA; ^2^ Department of Neurology The Johns Hopkins School of Medicine Baltimore Maryland USA; ^3^ Department of Neuroscience and Cognitive Science The University of Maryland College Park Maryland USA; ^4^ Department of Psychology The Johns Hopkins School of Medicine Baltimore Maryland USA; ^5^ Department of Biology The University of Maryland College Park Maryland USA

**Keywords:** cognition, functional connectivity, magnetoencephalography, recovery, stroke

## Abstract

**Objective:**

Patients with minor stroke exhibit slowed processing speed and generalized alterations in functional connectivity involving frontoparietal cortex (FPC). The pattern of connectivity evolves over time. In this study, we examine the relationship of functional connectivity patterns to cognitive performance, to determine neurophysiological underpinnings of improvement, and whether connectivity profiles may be useful in evaluating and predicting longer‐term cognitive outcomes.

**Methods:**

Patients hospitalized with a minor ischemic stroke (NIH Stroke Scale < 10) were neurologically evaluated approximately 1 month following discharge. A battery of neuropsychological tests was administered to assess performance across multiple cognitive domains. Functional connectivity was evaluated using resting state magnetoencephalography (MEG). Repeat evaluations were performed 3–6 months later. The Network Localized Granger Causality framework was used to estimate functional connectivity at each visit. Relationships between functional connectivity and cognitive performance at each visit were assessed using cluster‐based permutation tests and mixed effects modeling.

**Results:**

Forty‐nine patients had available data for both follow‐up visits. The average age was 62.4 years; 57% were female; 39% were Black. Mixed effects models indicated significant increases in contralesional frontoparietal beta‐band connectivity across visits that corresponded to improved behavioral performance. Early reliance on the contralesional hemisphere was associated with better scores at visit 1, and continued reliance on areas within the ipsilesional hemisphere was associated with poorer performance at visit 2.

**Discussion:**

Specific connectivity profiles are associated with better acute and longer‐term cognitive performance and may indicate greater potential for recovery. Further studies are needed to determine if patterns are modifiable.

## 1 Introduction

Stroke is a leading cause of global mortality and acquired disability [1]. Although improvements in treatment and recovery have reduced age‐standardized death rates and improved patient outcomes, the estimated global cost remains high [2]. Advances in acute therapies for ischemic stroke such as intravenous thrombolysis and mechanical thrombectomy [3, 4] have significantly reduced the volume of infarct so that more patients are being discharged with smaller, so‐called minor strokes (NIHSS score < 10), fewer neurological deficits, and better potential for recovery [5, 6]. Patients with minor stroke commonly endorse difficulties with attention, focus, and processing speed [7]. In most cases, infarcts are small and subcortical. As areas of eloquent cortex are not involved, alternative explanations must be considered regarding the underlying pathophysiology of deficits. Our prior work [8] suggests that minor strokes result in a generalized disruption of brain networks involving cortical areas that are not directly affected by the stroke. These network changes manifest electrophysiologically and can be studied using functional connectivity analysis applied to magnetoencephalography (MEG) recordings of neural activity [8, 9, 10].

Magnetoencephalography is a non‐invasive recording technique that measures magnetic fields generated by electrical neural activity in the brain. MEG bears similarities to the more prevalent electroencephalography (EEG), but with more accurate spatial localization of the underlying neural activity, since magnetic fields propagate through tissue layers largely undistorted. The millisecond temporal resolution of MEG further makes it an ideal tool to investigate differences in processes involving rapid and distributed neural signal propagation underlying cognitive processing.

This study was designed to explore the relationship between functional connectivity and cognitive performance throughout stroke recovery. Behavioral measures of cognitive performance, such as the Montreal Cognitive Assessment (MoCA), are commonly administered to patients post‐stroke. Identifying neural correlates of these behavioral measures using neuroimaging serves to improve our understanding of the mechanisms underlying recovery and may provide a predictive tool or surrogate measurement of response to treatment. While the cognitive dysfunction endorsed by patients with minor stroke appears clinically to best localize to the frontal lobe, MEG‐based investigations suggest that it may actually be due to a more generalized disruption of functional connectivity, independent of lesion size or location [8, 11]. MEG‐derived biomarkers can also be used (1) to assess whether any intervention has the desired effect on intermediate steps, in this case cognitive circuits, irrespective of behavioral outcome, and (2) as a potential predictor of response to therapy prior to the initiation of treatment. The use of MEG also allows us to observe the relationships between discrete areas of the brain over time to determine if specific patterns of recovery correspond to better clinical outcomes.

Results will allow not only for a better understanding of the relationship between functional connectivity and cognitive impairment after stroke, but will also elucidate the neurophysiological underpinnings of stroke recovery and confirm the potential use of MEG to derive biomarkers for future clinical trials.

## 2 Methods

### 2.1 Standard Protocol Approvals, Registrations, and Patient Consents

Following hospitalization for acute ischemic stroke, patients were scheduled for outpatient clinic follow‐up approximately 1 month after discharge. The stroke clinic provides the infrastructure to follow patients over time and enroll them in ongoing longitudinal studies and clinical trials. Patients are evaluated at consistent time points: 1, 3–6, 12, and 24 months post‐hospitalization using clinical examinations, neuropsychological batteries, functional scales, neuroimaging, and patient‐reported outcomes. All participants provided their written informed consent, and the study was approved by the Johns Hopkins Institutional Review Board. At their initial baseline visit, each participant completed a neurological assessment and was screened to determine their suitability for study participation. Those meeting inclusion criteria were scheduled for their baseline neuroimaging to evaluate functional connectivity.

### 2.2 Inclusion and Exclusion Criteria

**FIGURE 1:**
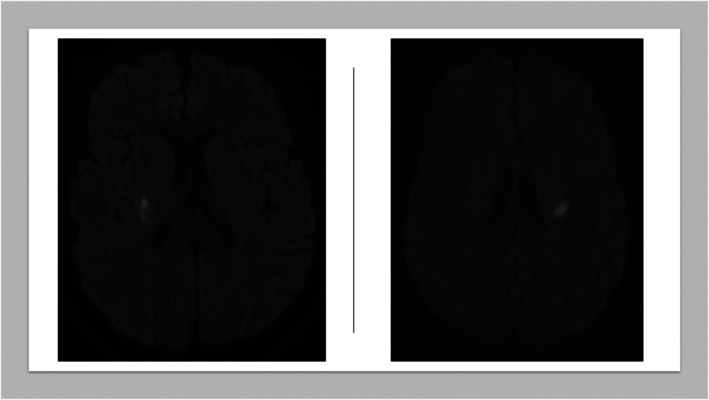
Two representative diffusion weighted MRI images of minor stroke. Note the small size and lack of involvement of eloquent cortex.

Forty‐nine patients were determined to be appropriate candidates for study inclusion. All were adults (≥ 18 years) who had experienced their first ever clinical supratentorial ischemic stroke and presented with an admission NIH Stroke Scale score of < 10. This cutoff avoided the inclusion of patients with large vessel territory involvement (e.g., M1 or M2 occlusion) and those with significant hemiparesis, aphasia, or hemispatial neglect that may confound outcomes, allowing the study to focus on the recovery of cognitive deficits. However, the mean NIHSS score of the cohort was in reality only 2.8 (SD 2.2), with an average infarct volume of just 4.5 cc (see Figure 1 for representative examples of infarct sizes and locations). Those without an MRI or who had no abnormality on diffusion‐weighted imaging (consistent with a transient ischemic attack (TIA) or diffusion‐negative stroke) were excluded. Candidate participants were also required to have had a good pre‐stroke baseline (modified Rankin Scale (mRS) of two or less) and no history of dementia or psychiatric illness, and the ability to return to the clinic for follow‐up evaluation. If the patient had another stroke at any point prior to completion of the study, they were excluded from further participation and their collected data censored from the subsequent analysis.

### 2.3 Clinical Measures and Cognitive Assessment

Data were collected regarding patient demographics (age, sex, self‐identified race, and education), stroke characteristics (admission and discharge NIHSS [12] score, infarcted hemisphere, lesion volume, and cortical versus subcortical location), functional baseline (pre‐stroke mRS [13] score), and medical comorbidities (history of smoking, hypertension, diabetes, depression, and Charlson Comorbidity Index [14]). A brief cognitive battery of tests was administered to assess for cognitive dysfunction [15]. For this analysis, we focused on performance on the Montreal Cognitive Assessment (MoCA) at each visit as it allows for a global measure of cognitive function that incorporates multiple cognitive domains into a single score and can be followed over time.

### 2.4 MEG Recording

Baseline and follow‐up neuroimaging was also performed to evaluate changes in functional connectivity using magnetoencephalography (MEG). Data were collected using a 157 axial gradiometer whole head KIT (Kanazawa Institute of Technology) MEG system with subjects resting in the supine position in a magnetically shielded room (Vacuumschmelze GmbH & Co. KG, Hanau, Germany). Participants were asked to fixate on a cross projected onto an overhead screen within the room; for the resting‐state task, no audio or visual stimulus was provided. Three minutes of resting‐state data were recorded per participant in two 1.5‐min blocks at a sampling rate of 1 kHz with an online 200 Hz lowpass filter and 60 Hz notch filter.

Prior to data collection, the head shape of each subject was digitized using a Polhemus 3SPACE FASTRAK system, and head position within the scanner was measured using five marker coils. The marker coil locations and digitized head shape were used to register the subject with the FreeSurfer [16] “fsaverage” brain template using an affine transformation.

### 2.5 Data Pre‐Processing

The MEG data were preprocessed using the mne‐python [17] (version 1.3.1) and eelbrain [18] (version 0.38.5) packages. To avoid edge effects, the first and last 15 s of data were dropped from each segment, resulting in two non‐abutting, 60‐s segments of data per subject. Bad channels were excluded, and temporal Signal Space Separation (tSSS) was used to filter out magnetic fields originating from outside subjects' brains. Independent Component Analysis was then performed to remove artifacts caused by heartbeats and eye blinks. Data were subsequently filtered to the beta band (13 to ~25 Hz) using a minimum‐phase FIR bandpass filter. The beta band was chosen based on prior work showing significant abnormalities in those with minor stroke [19]. Finite impulse response bandpass filters with negligible leakage were employed to ensure that border effects in the frequency domain were minimized. A downsampling frequency of 50 Hz was chosen to both include the upper end of the beta band (just under 25 Hz) and reduce the runtime of the algorithm.

### 2.6 Functional Connectivity

The Network Localized Granger Causality (NLGC) framework was used to estimate directional cortical connectivity at each visit [20]. NLGC uses a statistical modeling procedure to estimate Granger Causal (GC) directed links between regions of cortex directly from MEG measurements. The resulting map of connections produced by NLGC spans the entire cortex divided into 84 cortical patches, centered at vertices in the ico‐1 source space. Each cortical patch is represented by 4 leading principal components that summarize activities from the finer ico‐4 source space (four components were chosen as a tradeoff between source estimation accuracy and computational complexity) [20]. The NLGC models are fit with an order parameter that is determined for each subject by comparing Akaike Information Criterion (AIC) values for a variety of candidate orders and picking the lowest one. Order 2 models were used because they best fit the majority of subjects.

### 2.7 ROI Selection

NLGC analysis was applied to each participant's resting‐state MEG data from each visit. The resultant connectivity maps spanned 84 patches tiling the cortical mantle. After a lateralization test revealed no significant differences between right and left hemisphere strokes for these measures (see Statistics: whole‐brain lateralization and Results: analysis 1), participants' right hemispheres were warped to mirror the left hemisphere patches, and individuals with right hemisphere strokes had their brains reflected. These 84 patches were mapped onto anatomical regions of interest (ROIs) in each hemisphere. The anatomical ROIs were selected based on their putative functional roles, and their borders were delineated using the Desikan‐Killiany atlas. For instance, all patches within the temporal cortex were assigned to either medial or lateral temporal cortex anatomical regions, which are thought to be functionally distinct. We chose 11 anatomical regions in each hemisphere—22 total regions—similar to those defined by Marenco et al. [21] Of particular interest is the sensorimotor region surrounding the frontoparietal cortex (FPC) where abnormalities in the beta band have been previously demonstrated in minor stroke patients [19]. For this reason, the FPC (comprising Desikan‐Killiany [22] labels “precentral”, “postcentral”, “paracentral”) was assigned its own anatomical region among the 22.

### 2.8 Statistics

Mixed‐effects models were employed to characterize changes in observed link counts between regions of interest and evaluate the contribution of potential confounders including age, race, sex, highest level of education, and lesioned hemisphere. Mixed‐effects modeling is a regression framework that includes both fixed and random effects terms. Fixed effects treat observations as increasing or decreasing commensurately with a predictor of interest (e.g., a person's income may increase with age), whereas random effects model uncertainty in the observations that do not arise systematically but are instead the product of variability in certain groups within the data (e.g., within‐subject or within‐trial variability). In our study, GC link counts were modeled using a family of Poisson distributions, which were chosen to conform to empirical distributions of links obtained from our data. During trials with low signal to noise ratio (SNR), NLGC obtains a lower hit rate, resulting in a lower number of detected links in some trials. Because initial models underfit these zeros, the models ultimately used allow for zero inflation (zero values modeled by a separate point‐mass distribution at zero).

Mixed effects models were employed in every analysis, though the scope of the data to which they were applied varied. In the case of whole‐brain analysis, one model was fit to each connected ROI pair, necessitating a method of false discovery correction detailed below. Narrower scopes modeled four regions of interest simultaneously and so did not require multiple comparison correction.

### 2.9 Analysis

The investigation was structured into two analysis phases, increasing in level of granularity. The first analysis was conducted at the level of the whole‐brain and was intended to identify lateralization of beta‐band connected networks as well as the networks expressing the strongest differences between patients with better and worse cognitive scores. The second analysis focused on the ROI determined by the whole‐brain analysis and relates the connectivity data to clinical data and recovery patterns.

#### 2.9.1 Analysis 1: Whole‐Brain Connectivity

**FIGURE 2:**
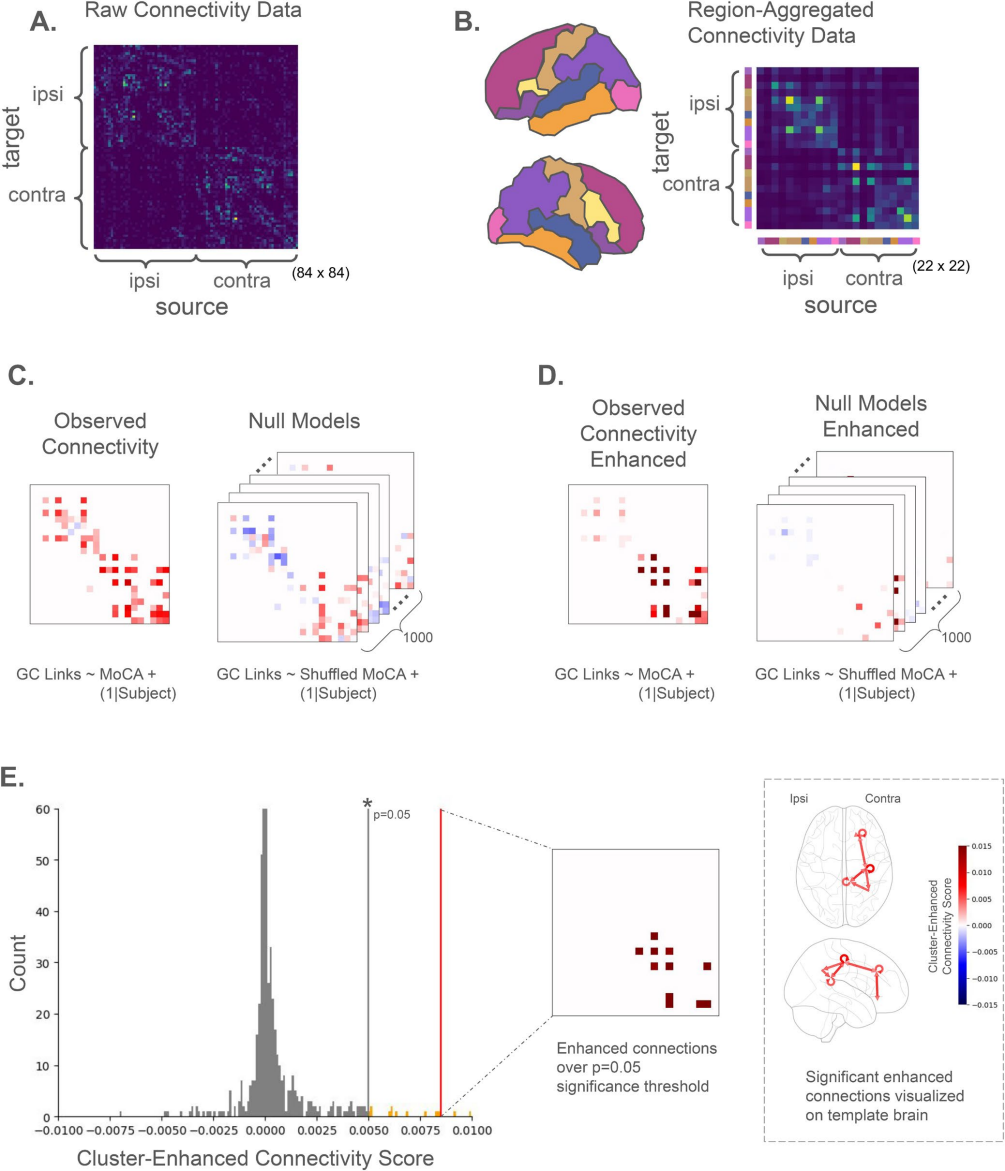
Whole‐brain testing protocol used to analyze links that demonstrate consistent network‐level effects. (A) Grand‐average connectivity data in subjects' native source space (ico‐1). (B) Connectivity data from (A) after aggregation within each anatomical region of interest. Anatomical region boundaries are obtained from the Desikan‐Killiany atlas and correspond to the colored regions on the brain inset. (C) Mixed‐effects regression models are fit to each set of connectivity links (grid cell) in (B) and the posterior mean value for each fixed‐effect coefficient is plotted. Darker red or blue cells indicate more positive or negative coefficient values respectively. Regression models were also refit 1000 times with the fixed‐effect predictor shuffled between subjects to form a set of null‐distribution (“null”) coefficient values for each grid cell. (D) Connected networks that share consistent, but possibly weak, effects are enhanced using the nbs‐TFCE algorithm. Notice how some consistent effects from (C) are enhanced—appear darker—after nbs‐TFCE is applied. In contrast, the null models share fewer consistent effects and are therefore minimally enhanced. (E) The largest enhanced values in the observed data are compared to the largest enhanced values across all 1000 null models. Only effects that are larger than 95% of all observed effects in the null models are deemed significant.

The number of beta‐band GC connections between each of the 22 whole‐brain anatomical regions was summed, resulting in a single 22 × 22 connectivity matrix for every trial. A mixed effects model was fit to each grid cell across connectivity matrices, representing the set of directed links connecting a pair of regions. Each regression models a fixed effect component that, on its own, captures small effects in the data, not enough to pass false discovery correction. However, because each link is one connection within a larger encompassing network, these small effects would, in aggregate, form a large significant effect at the network level. These networks can be thought of as a cluster to which membership is determined by connected graph components. This is analogous to spatial clusters of neural activity in fMRI voxel analysis for which it is common to use a cluster's membership to “enhance”—boost the signal value of—its constituent voxels. This enhancement is often performed using a robust nonparametric technique called Threshold‐Free Cluster Enhancement (TFCE) [23] that takes a weighted combination of cluster breadth at many signal thresholds. In our analysis, we use network‐based‐statistic TFCE (nbs‐TFCE) [24, 25, 26, 27], a variant of TFCE designed for network graph structures, to enhance our directional connectivity networks. The statistic value enhanced by nbs‐TFCE is the fixed effect coefficient of each regression. We correct for false discovery and assess the significance of our nbs‐TFCE enhanced networks using a two‐sided max‐statistic cluster permutation test with 1000 permutations. Figure 2 illustrates whole‐brain analysis workflow.

##### 2.9.1.1 Lateralization

Whole‐brain tests were first used to assess lateralization differences between left and right hemisphere strokes. A single zero‐inflated Poisson model was fit to each grid cell (*i*, *j*) across the connectivity matrices:

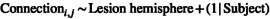

where (1|Subject) is the notation used to denote a per‐subject random intercept to model baseline connectivity per participant. These observed networks were enhanced using nbs‐TFCE with *E* = 0.75 and *H* = 3.25 [25]. One‐thousand shuffles of Lesion Hemisphere labels were performed across subjects, and models were refit to these shuffles for each link. The observed model enhanced coefficients were compared to the largest null model enhanced coefficient for each fold to obtain a monte‐carlo *p*‐value for each observed effect.

##### 2.9.1.2 MoCA Scores

Next, mixed‐effects models were used to assess network changes on the basis of patients' MoCA scores. The first analysis tested the relationship between patients' connectivity observations and MoCA scores at the first follow‐up visit (Connectivity_visit 1_ ~ MoCA_visit 1_ + (1|Subject)). The second tested the relationship between patients' connectivity observations and MoCA at the second follow‐up visit (Connectivity_visit 2_ ~ MoCA_visit 2_ + (1|Subject)). The third tested whether participants' connectivity networks at their first follow‐up could predict (1) cognitive improvement, or change in MoCA score between visits (Connectivity_visit 1_ ~ (MoCA_visit 2_—MoCA_visit 1_) + (1|Subject)), and (2) future performance, or overall performance at visit 2 (Connectivity_visit 1_ ~ MoCA_visit 2_ +  (1|Subject)).

#### 2.9.2 Analysis 2: Frontoparietal Cortex (FPC) Connectivity

The change of FPC connectivity throughout recovery was evaluated. Ipsilesional and contralesional FPC regions were compared to non‐FPC regions, resulting in a 4 × 4 matrix of connections. Linear mixed‐effects models were fit using a systematic, reproducible approach based on the buildmer package [28] (version 2.11). This approach was not undertaken for the whole‐brain models because of the computational demands of whole‐brain permutation testing.

The buildmer procedure begins by selecting a maximal model as a target. The maximal model incorporates many fixed and random effects but is not expected to converge. Instead, smaller models are “built toward” the maximal model in an iterative, model building procedure whereby fixed and random effects are added, one at a time, until the model fails to converge. Then, terms in this maximal feasible model are sequentially pruned until only statistically significant terms remain, resulting in the final, optimal model. This maximal model was selected to be:

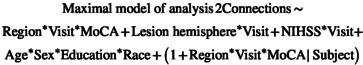




The final buildmer model extracted the significant factors that were central to the observed changes in FPC connectivity. Post hoc differences among the levels of the model's effects were tested using pairwise comparisons based on estimated marginal means, with Holm corrections using the package emmeans [29] (version 1.10.4).

## 3 Results

The average age of the cohort was 62.4 years. Thirty‐nine percent were black; 57% were male. For a full description of patient characteristics please see Table 1. All participants underwent both clinical and neuroimaging evaluation at both time points. Clinically, most patients improved between visits 1 and 2. Radiographically, there was also significantly increased connectivity in 11 of 16 region pairs (Bonferroni *p* < 0.05, see Table S1).

**TABLE 1:** Patient characteristics.

	Total (*N* = 49)
Demographics	
Age, mean years (SD)	62.4 (13.9)
Sex, % male	57%
Race, % black	39%
Education, mean years (SD)	13.7 (2.4)
Premorbid IQ, mean (SD)	109.0 (10.9)
Handedness, % right	88%
Occupation code, %	
1—Professional	17%
2—Intermediate	22%
3—Skilled	22%
4—Semiskilled	29%
5—Unskilled	10%
Stroke characteristics	
Admit NIHSS, mean points (SD)	2.8 (2.2)
Stroke volume, mean cc (SD)	4.5 (7.2)
Hemisphere, % left	47%
Subcortical, % yes	55%
Discharge NIHSS, mean points (SD)	1.3 (1.5)
White matter grade—CHS, %	
1	23%
2	48%
3	9%
4	9%
5	5%
6	5%
7	0%
8	2%
Stroke etiology, %	
Large vessel	22%
Cardioembolism	11%
Small vessel	46%
Other determined etiology	2%
Undetermined etiology	20%

### 3.1 Analysis 1: Whole‐Brain Connectivity

**FIGURE 3:**
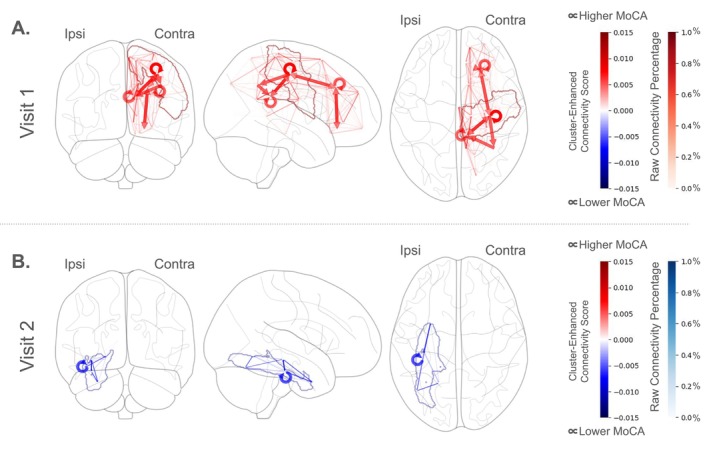
Significant network differences between stroke patients based on their cognitive scores at each visit. Large arrows indicate the directions where increased aggregate connectivity is significantly associated with MoCA score (looped arrows indicate local connectivity within a region). Red colors indicate a positive association between connectivity and MoCA and blue colors indicate a negative association. (A) At patients' first visit, increased contralesional connectivity involving frontoparietal areas was associated with better MoCA scores. (B) At their second visit, three to 6 months post‐stroke, individuals with better MoCA scores presented with less connectivity in ipsilesional temporal cortex, namely in parahippocampal, entorhinal, and fusiform areas, which are associated with memory and temporal perception. Both panels: Small colored arrows display the raw data underlying the gross connectivity plotted in large arrows. The region with the largest effect at each visit is outlined and colored accordingly. In (A), contralesional frontoparietal regions outlined in red correspond to Desikan labels “precentral”, “postcentral”, and “paracentral”; in (B), ipsilesional temporal regions outlined in blue correspond to Desikan labels “parahippicampal”, “entorhinal”, and “fusiform”.

The first functional connectivity analysis was conducted within the beta band and across the whole brain at patients' first and second follow‐up visits. Patients' functional connectivity maps were predicted from their intake MoCA scores at both timepoints (Connectivity_visit 1_ ~ MoCA_visit 1_ + (1|Subject) and Connectivity_visit 2_ ~ MoCA_visit 2_ + (1|Subject)) and regions with a significant relationship between MoCA and connectivity were obtained using the nbs‐TFCE procedure. Significant effects are plotted in Figure 3. At patients' first visit, we observed significantly more contralesional connectivity (monte‐carlo *p* = 0.014) involving frontal regions and FPC areas (red arrows) in patients with better MoCA scores. At patients' second visit, individuals with better MoCA scores displayed significantly less connectivity (monte‐carlo *p* = 0.04) in ipsilesional regions of temporal cortex, namely in parahippocampal, entorhinal, and fusiform areas (blue arrows). These areas are associated with memory and temporal perception. First‐visit connectivity was not predictive of overall cognitive performance at 6 months (monte‐carlo *p* = 0.18). However, there was a large, though not quite significant, negative effect (monte‐carlo *p* = 0.051) in contralesional hemisphere areas similar to those identified at the first visit associated with change in MoCA score, suggesting those who had not yet recruited the contralesional hemisphere may have potential to do so and improve.

### 3.2 Analysis 2: FPC Connectivity

Using buildmer model selection to systematically fit the mixed effects model to the FPC connectivity data within the beta band, the initial best‐fit model after pruning was given by the following formula, where each term significantly contributed to the model fit (i.e., removal of any term would significantly harm the explained variance of the model):

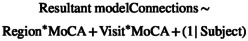




The result of the model's significant predictors is that the FPC connectivity varies depending on the brain region, visit, and importantly, the individual's cognitive ability measured by the MoCA at the time of each visit. The significance of the interaction between region and MoCA indicates that those with better MoCA scores have a different regional connectivity pattern than those with more cognitive impairment. The random intercept indicates that the predictions maintain their significance even when accounting for individual differences in baseline connectivity.

**FIGURE 4:**
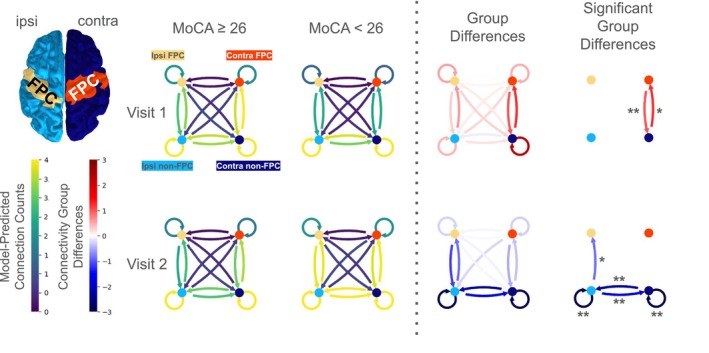
Ipsilesional and contralesional connectivity involving FPC regions at both visits. Connectivity is contrasted between patients whose cognitive scores at their first visit were below the “clinically normal” MoCA cutoff of 26 and those who exceeded the cutoff. Notice that at the first visit, the MoCA < 26 group has more connectivity involving the ipsilesional cortex. In contrast, the clinically normal MoCA group uses relatively more contralesional cortex at their first visit. By their second visit, all areas see a relative increase in connectivity, and FPC connectivity patterns begin to converge. *p < 0.05, **p < 0.005.

The predicted connectivity differences between clinically normal and abnormal MoCA groups are shown for each region and visit in Figure 4. At patients' first visit, increased contralesional connectivity involving FPC is significantly associated with better MoCA scores (Contra ALL → Contra FPC, *p* = 0.02; Contra FPC → Contra ALL, *p* = 0.009; see Table 2). At their second visit, patients with better MoCA scores consistently show less connectivity involving ipsilesional cortex—four of the five significant connections involve non‐FPC ipsilesional areas as either a connectivity source or target (see Table 2). This is consistent with persistent reliance on ipsilesional cortex, hindering long‐term recovery outcomes. Model predictions for each region were obtained using the emmeans package, enabling “marginalization” over random effects to assess the main effects of interest.

**TABLE 2:** Significant differences between patients with clinically normal and abnormal (MoCA < 26) scores.

Regions	Visit	Estimate	Standard error	*Z* ratio	Bonferroni *p* value
Contra ALL → Contra FPC	1st	1.317	0.574	2.294	0.022
Contra FPC → Contra ALL	1st	1.644	0.634	2.593	0.010
Contra ALL → Contra ALL	2nd	−5.682	2.072	−2.742	0.006
Contra ALL → Ipsi ALL	2nd	−2.223	0.796	−2.792	0.005
Ipsi ALL → Contra ALL	2nd	−3.014	0.919	−3.280	0.001
Ipsi ALL → Ipsi ALL	2nd	−9.013	2.398	−3.758	< 0.001
Ipsi ALL → Ipsi FPC	2nd	−1.515	0.635	−2.385	0.017

*Note:* Comparisons were made for connectivity between each region (FPC vs. all other areas) at each visit and were Bonferroni‐corrected for multiple comparisons.

## 4 Discussion

Our results suggest that neural connectivity is directly linked with cognitive performance at each phase of stroke recovery, and, more importantly, continues to evolve over time in a predictable and necessary way. Patients who present initially with better cognitive function exhibit increased connectivity in the contralesional FPC at their first follow‐up visit, only weeks after their stroke, compared to those who are more impaired. By their second follow‐up visit, 3–6 months later, the majority of individuals demonstrate generalized increases in connectivity bilaterally consistent with the positive trajectory in clinical symptoms traditionally observed during this time as part of the natural history of stroke recovery. However, those who score best on the MoCA continue to show a relative mobilization of connectivity away from ipsilesional areas compared to others. Most notably, this later transfer occurs in areas outside of FPC, particularly regions within temporal cortex. Taken together, these results point to evolving compensatory mechanisms that initially shift connectivity to homologous areas within the non‐lesioned hemisphere, but eventually attempt reintegration of the damaged side. Furthermore, reliance on alternative areas of higher‐level function within ipsilesional cortex without adequate recruitment of the contralesional side may represent a maladaptive response associated with delayed improvement and poorer cognitive performance long term.

Both baseline connectivity and patterns of recovery differ significantly between individuals displaying better performance on the MoCA than those with abnormal scores indicating more severe cognitive dysfunction. Patients with higher scores by our cutoff fell into a “clinically normal” range of performance on the MoCA (26 or greater). However, it is important to note that a clinically normal score does not mean it is reflective of an individual's baseline, nor that they were not affected by their stroke, as all patients endorsed cognitive symptoms at the time of assessment and the majority improved in score by their subsequent follow‐up visit. Using a cutoff of 26 to define groups simply allowed us to investigate differences in functional connectivity that could be associated with behavioral outcomes by stratifying by level of performance. In fact, it effectively revealed that initial transfer of connectivity away from the lesion to the non‐affected hemisphere followed by the subsequent reincorporation of previously damaged brain is associated with better performance at each time point. These findings have significant ramifications for our understanding of post‐stroke recovery.

Neural reorganization after stroke has been widely observed secondary to numerous types of central nervous system pathology. Most relevant to our work, Hillis and others have described reorganization of language to both perilesional regions and homologous areas within the opposite hemisphere following stroke [30]. One hypothesis is that lesion size may play a role in determining the extent of reorganization. If the infarct volume is too large, function has no choice but to cross over to contralesional cortex, which may be more expensive from a recovery standpoint, and may or may not result in a better long‐term outcome. Our study includes only individuals with small strokes, where forced contralesional reorganization should not occur. Therefore, our findings suggest not only that other factors may influence network reorganization, but that, at least for small infarcts, recruitment of the contralesional undamaged hemisphere followed by eventual re‐engagement of injured regions may be required for the best clinical outcome.

Our results also indicate that the specific areas of engagement that are important for optimal cognitive recovery change with time. Individuals performing better at visit 1 displayed a greater degree of contralesional connectivity involving specifically the contralesional FPC, indicating the early transfer of function away from the damaged hemisphere, potentially to homologous areas on the opposite side. In contrast, individuals with demonstrable levels of cognitive impairment showed comparatively more ipsilesional connectivity, theoretically indicating continued reliance on damaged brain regions or perilesional areas traditionally responsible for other functions. These findings corroborate previous studies. Westlake et al. [26] observed similar MEG‐derived functional connectivity patterns in the alpha band during the subacute phase of recovery, while Soleimani et al. [8] compared a small cohort of minor stroke patients to normal controls and also found reduced ipsilesional connectivity within the beta band present during the first month after stroke, with a relative increase in connectivity in the contralesional cortex. We chose to focus on the beta band, particularly within the FPC, based on this and other prior work showing its alteration in motor deficits and processing speed after stroke. While beta‐band disruptions have been long reported in patients with motor impairment, Kulasingham et al. [19] demonstrated that patients with minor stroke and only abnormal processing speed but no significant hemiparesis also showed significant abnormalities. The reduced bilateral rolandic beta activity during the recovery period, irrespective of lesion location (and most notably with strokes outside of the motor pathway), suggests that small and even distant lesions are able to result in global network impairment. It is important to note that those beta power abnormalities persisted regardless of clinical improvement, illustrating that power within the beta band alone is not driving behavior, or responsible for the continued evolution of directional functional connectivity, and highlighting the need for additional studies such as this one to better understand the more nuanced, adaptive or maladaptive reorganization patterns associated with performance.

By visit 2, all of our patients saw some degree of re‐integration of the ipsilesional frontoparietal regions within the network. However, individuals with persistent cognitive impairment displayed increased levels of connectivity in ipsilesional regions *outside* of the FPC. It may initially appear unclear whether this indicates an ongoing failure to fully mobilize activity to areas in the non‐lesioned hemisphere or instead a preemptive re‐integration of damaged areas of cortex. In Soleimani et al.'s cohort [8], patients were followed up to 12 months after infarct, demonstrating increased generalized functional connectivity between 1 and 6 months with a subsequent pruning back of connections at later time points. Siegel et al. [27] observed a similar inflection point in resting‐state fMRI connectivity from some of their participants, though functional connectivity increased linearly toward control levels for most. These differences are possibly explained by differences in stroke severity between cohorts and inherent differences between electrophysiological and hemodynamic functional connectivity. What both studies seem to suggest, is that recruitment of the contralesional hemisphere through increased interhemispheric connections must first occur before the ipsilesional hemisphere can begin reintegration and traditional cognitive circuits ultimately repaired. It follows that in this study, we are most likely observing patients at 6 months with impaired MoCA scores who have not yet been able to increase their reliance on the contralesional hemisphere and are instead using perilesional regions to a larger degree. Future evaluation of the cohort is needed to confirm if there is a delayed shift back to the damaged hemisphere in these patients.

After illustrating that particular patterns of connectivity are associated with cognitive performance at specific time points, the next logical question is whether the initial visit can predict degree of recovery and future performance. We see some evidence for this. Patients who improve the most between visits 1 and 2 initially show *less* contralesional recruitment of the same areas demonstrating *increased* activity in those with better MoCA scores at visit 1. In other words, their pattern is consistent with those with poorer initial performance. It follows that the finding is more likely a function of their capacity for change (i.e., by having lower initial scores they have more room to improve by the second visit) than a true predictor for improvement. Importantly, we did not find an association between the pattern of connectivity at visit 1 and cognitive performance at visit 2. This may be due to the variability of connectivity between patients, the relatively minor cognitive deficits of the cohort in general, or that the majority of patients demonstrated at least some degree of recovery between visits. Future work evaluating differences between individuals with more severe impairment and larger differences in MoCA scores at each time point is a critical next step.

This study has some limitations. The cohort was relatively small, raising the possibility it was underpowered to detect small differences in functional connectivity, particularly when clinical performance began to converge by visit 2. In addition, there was no formal control group without infarct. Nevertheless, results are consistent with those of Soleimani et al. [8], where the observed evolution of connectivity over the first 6 months of stroke recovery in both studies was consistent. Finally, while mixed effects models were used to account for some factors that might influence both connectivity patterns and clinical recovery (e.g., age), additional factors leading to individual variability may not have been accounted for such as the degree of co‐existing white matter disease. We chose not to include white matter burden in our model given that nearly 75% of our cohort had a CHS score of 2 or less (consistent with minor disease). A larger study including individuals with higher burdens of disease may allow for a more complete evaluation of its impact, as well as analysis of whether various connectivity profiles and involvement of specific neural circuits are associated with impairment in specific cognitive domains using a more extensive neuropsychological battery.

Despite these limitations, the data show that distinct patterns of beta‐band connectivity, specifically involving the FPC region, are linked to overall cognitive performance at predictable points during stroke recovery and lay the groundwork for future studies. The study further suggests that early patterns of connectivity may suggest longer‐term recovery potential, and raises the possibility that connectivity profiles may be useful in predicting who may benefit from targeted therapies to enhance connectivity. Whether network connectivity can be modified using other interventions such as neurostimulation remains to be seen and warrants further investigation.

## Author Contributions

Vrishab Commuri: data analysis, initial drafting of the manuscript. Isabella Dallasta: data acquisition, data analysis, review and editing of manuscript. Ciaran Stone: data acquisition. Sophia Girgenti: data acquisition, data analysis. Neda Gould: study conceptualization and design, data acquisition. Rafael H. Llinas: study conceptualization and design, review and editing of manuscript. Jonathan Z. Simon: study conceptualization and design, data analysis, review and editing of manuscript. Elisabeth Breese Marsh: study conceptualization and design, data analysis, review and editing of manuscript, study oversight.

## Funding

This work was supported by the National Institutes of Health (Grants R21 AG068802‐01 and RF1 AG079324).

## Conflicts of Interest

The authors declare no conflicts of interest.

## Supporting information


**Table S1:** Significant increases in connectivity between visits 1 and 2 for 11 of 16 region pairs.

## Data Availability

Scripts used for analysis are available in GitHub as described within the manuscript. Grouped data is available in aggregate on ClinicalTrials.gov. Source data is available for collaboration upon reasonable request from the PI.
