# Supplementary material for: Functional Connectivity Linked to Cognitive Recovery After Minor Stroke
Source: Ann Clin Transl Neurol. 2025 Dec 6;13(5):888–98. doi: 10.1002/acn3.70271 (PMC13161874; doi:10.1002/acn3.70271)
Supplement: Supplementary file 1 — Table S1: Significant increases in connectivity between visits 1 and 2 for 11 of 16 region pairs. [file ACN3-13-888-s001.docx]

**Supplemental Table S1.** Significant increases in connectivity between visits 1 and 2 for 11 of 16 region pairs.

| Regions | Difference Estimate | Standard Error | Z Ratio | Bonferroni P Value |
| --- | --- | --- | --- | --- |
| Contra ALL → Contra ALL | 2.242 | 0.756 | 2.967 | 0.003 |
| Contra ALL → Contra FPC | 0.431 | 0.187 | 2.306 | 0.021 |
| Contra ALL → Ipsi ALL | 0.701 | 0.229 | 3.063 | 0.002 |
| Contra FPC → Contra ALL | 0.430 | 0.200 | 2.148 | 0.032 |
| Contra FPC → Contra FPC | 0.229 | 0.104 | 2.207 | 0.027 |
| Ipsi ALL → Contra ALL | 0.871 | 0.267 | 3.263 | 0.001 |
| Ipsi ALL → Contra FPC | 0.116 | 0.051 | 2.257 | 0.024 |
| Ipsi ALL → Ipsi ALL | 2.786 | 0.828 | 3.365 | 0.001 |
| Ipsi ALL → Ipsi FPC | 0.520 | 0.181 | 2.877 | 0.004 |
| Ipsi FP → Ipsi ALL | 0.485 | 0.187 | 2.596 | 0.009 |
| Ipsi FPC → Ipsi FPC | 0.236 | 0.109 | 2.155 | 0.031 |
